# Supplementary material for: Profiling the Urobiota in a Pediatric Population with Neurogenic Bladder Secondary to Spinal Dysraphism
Source: Int J Mol Sci. 2023 May 5;24(9):8261. doi: 10.3390/ijms24098261 (PMC10178886; doi:10.3390/ijms24098261)

**Supplementary Figure 1.** Number of bacterial species identified in each of urine samples included in the study, according to the sequencing depth obtained for each sample.

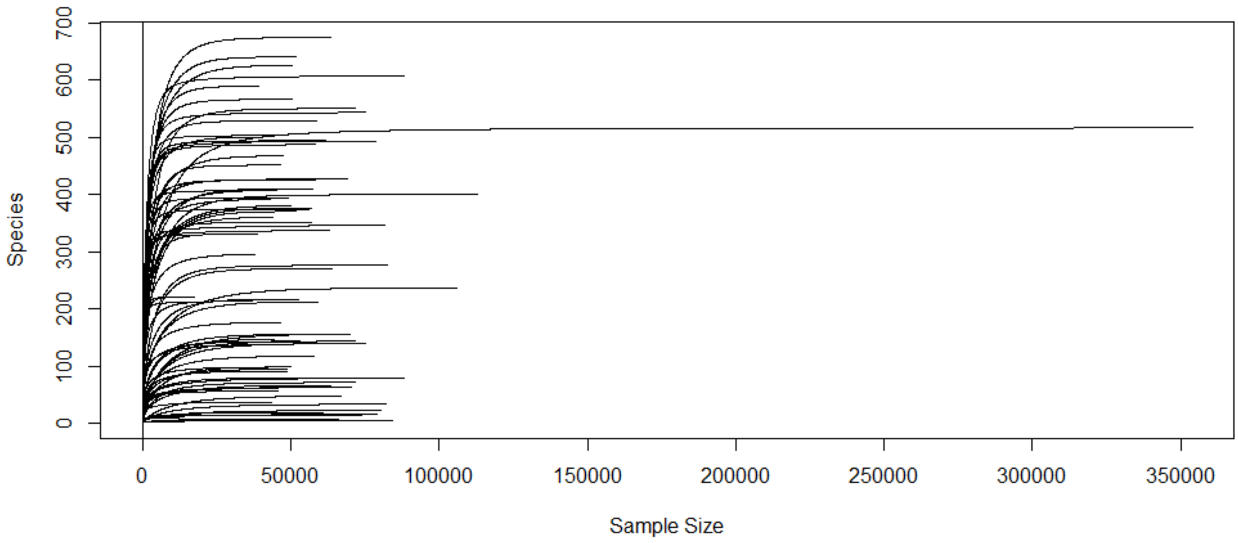


**Supplementary Figure 2.** Bar plot representation showing bacterial phyla in the study subjects' urine samples. In A, healthy controls are compared to SB patients. In B, SB patients who received CIC are compared with SB patients who did not receive CIC. Other phyla (i.e., with <1% relative abundance) include *Cyanobacteria*, *Fusobacteria*, *Lentisphaerae*, *Tenericutes*, *Synergistetes*, and *Verrucomicrobia*.

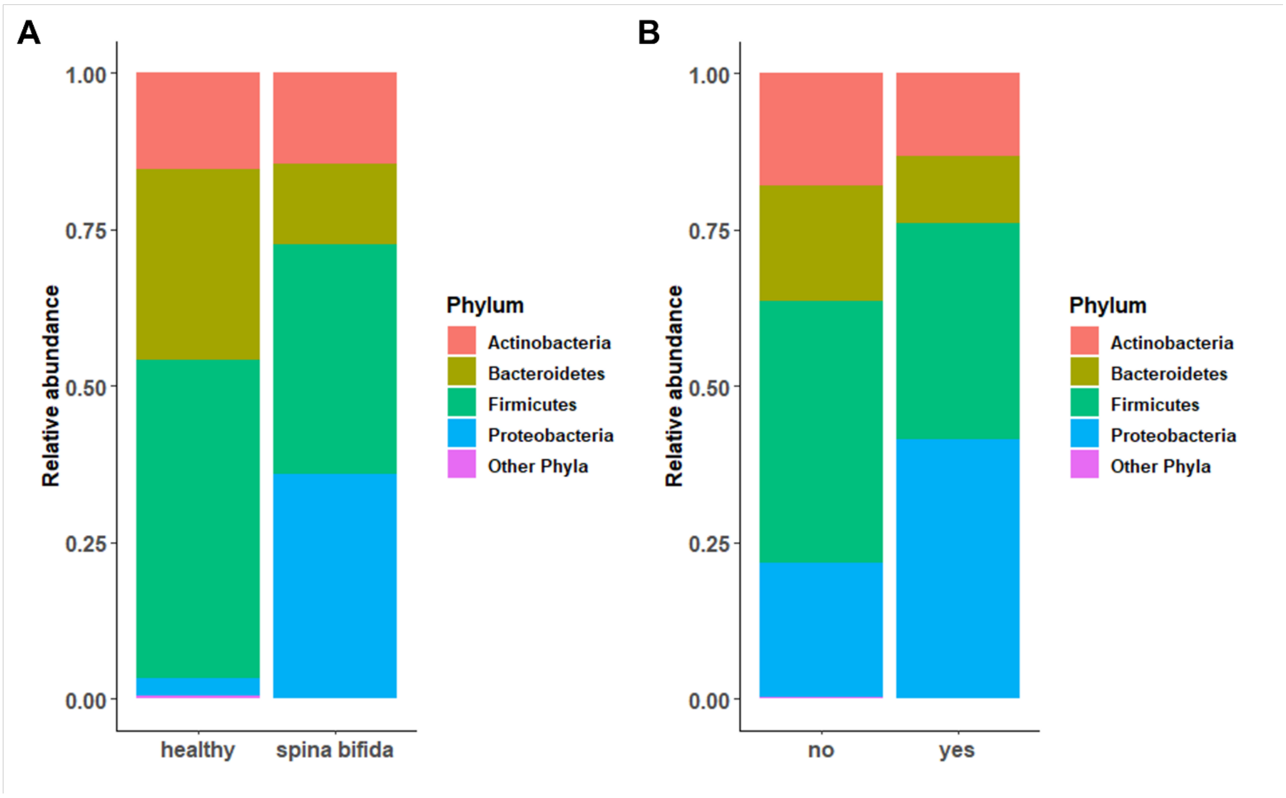

**Supplementary Figure 3.** Linear discriminant analysis effect size (LEfSe) results showing differentially abundant taxa between groups. In A, healthy controls are compared to SB patients. In B, SB patients who received CIC are compared with SB patients who did not receive CIC.

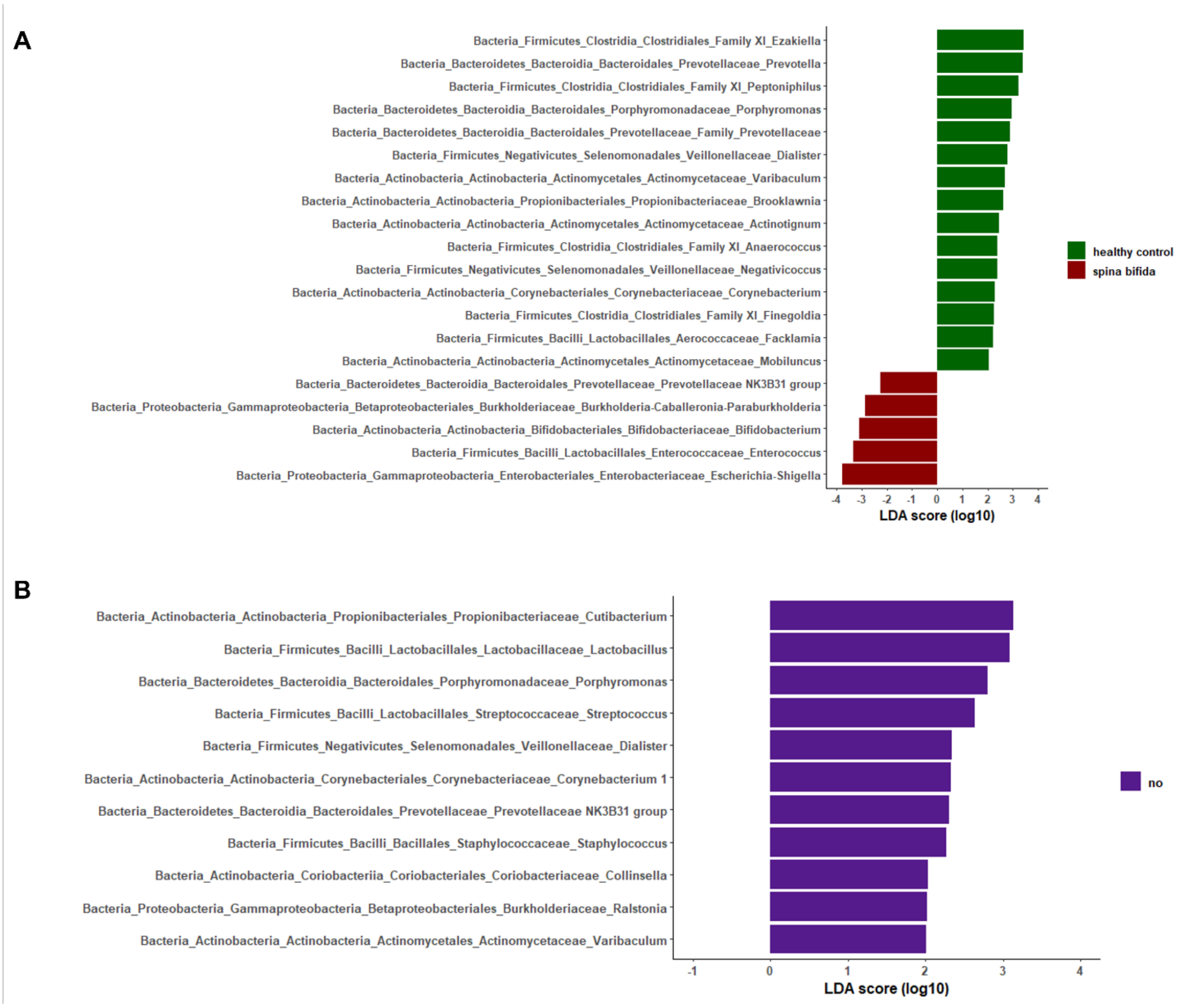

Supplement: Supplementary file 1 [file ijms-24-08261-s001.zip › ijms-2296461-supplementary.pdf]
